# Neuronal Regeneration after Electroacupuncture Treatment in Ischemia–Reperfusion-Injured Cerebral Infarction Rats

**DOI:** 10.1155/2017/3178014

**Published:** 2017-08-24

**Authors:** Shiang-Lin Liao, Yi-Wen Lin, Ching-Liang Hsieh

**Affiliations:** ^1^Graduate Institute of Acupuncture Science, College of Chinese Medicine, China Medical University, Taichung 40402, Taiwan; ^2^School of Post-Baccalaureate Chinese Medicine, College of Chinese Medicine, China Medical University, Taichung 40402, Taiwan; ^3^Research Center for Chinese Medicine & Acupuncture, China Medical University, Taichung 40402, Taiwan; ^4^Graduate Institute of Integrated Medicine, College of Chinese Medicine, China Medical University, Taichung 40402, Taiwan; ^5^Department of Chinese Medicine, China Medical University Hospital, Taichung 40447, Taiwan

## Abstract

Adult neuronal cells which can regenerate have been reported. The present study investigated whether acupuncture enhances neuronal regeneration in ischemic stroke rats. We established an ischemic stroke rat model by occluding the cerebral blood flow of the right middle cerebral artery for 15 minutes and then allowing reperfusion in Sprague–Dawley rats. The results indicated that, in these rats, 2 Hz electroacupuncture (EA) at both Zusanli (ST36) and Shangjuxu (ST37) acupoints reduced the infarction/hemisphere ratio 8 days after reperfusion and reduced the modified neurological severity score (mNSS) and increased the rotarod test time 4 and 8 days after reperfusion, respectively. In addition, 2 Hz reduced nestin immunoreactive cells in the penumbra area and the ischemic core area; 2 Hz EA also reduced Ki67 immunoreactive cells and increased glial fibrillary acidic protein immunoreactive cells in the penumbra area. These findings suggest that 2 Hz EA at the ST36 and ST37 acupoints has a neuroprotective role. However, additional studies are needed to further investigate these preliminary results.

## 1. Introduction

Ischemic stroke is a common type of stroke and a main leading cause of death, disability, and dependency worldwide [1]. Few therapies have proven effective for ischemic stroke, but they include the administration of intravenous recombinant tissue plasminogen activator (rt-PA) within 3 hours or the arterial injection of urokinase within 6 hours after stroke onset [2]. However, rt-PA is associated with the risk of bleeding and thus has both favorable and unfavorable consequences.

In Taiwan, acupuncture is a traditional Chinese medicine therapeutic strategy with a higher utilization rate in patients with stroke than in people without stroke [3]. One study found a lower risk of stroke in patients with traumatic brain injury who received acupuncture treatment than in those who did not receive acupuncture treatment [4]. Many other studies have demonstrated that acupuncture, which is a safe and effective treatment for stroke, can promote functional recovery in stroke patients without inducing any special side effects [5–8]. Our previous study indicated that acupuncture stimulation at the Baihui acupoint and four-spirit acupoints may induce an immediate effect that improves the balance function of stroke patients [9]. Moreover, 2 Hz electroacupuncture (EA) at the Baihui acupoint can reverse the middle cerebral artery occlusion- (MCAo-) induced behavior deficit and long-term potentiation [10].

Zhang et al. demonstrated that EA treatment improves the motor functions of the limbs and the activities of daily living in patients with hemiplegia caused by acute cerebral infarction; they also noted that this effect was associated with reduced serum levels of neuron-specific enolase, soluble protein-100B, and endothelin [11]. EA pretreatment protects the brain from transient cerebral ischemic injury, and EA can alleviate cerebral edema following ischemia in rats [12, 13]. Our previous study showed that acupuncture stimulation not only increased dopamine levels in the right cerebral cortex and hippocampus in the chronic cerebral hypoperfusion rat model, but also increased dopamine levels in the cerebral cortex in the cerebral ischemia–reperfusion injury rat model [14]. Additionally, Peng et al. determined that EA treatment may protect the blood–brain barrier by regulating aquaporin-4 expression after a cerebral ischemia–reperfusion injury [15]. An increasing number of studies have also focused specifically on different acupoints and the mechanism underlying the protective effects of EA against cerebral ischemia [16]; for example, the Zusanli acupoint has been widely discussed [17].

Acupuncture at the Zusanli acupoint can increase cell proliferation in the dentate gyrus after transient global ischemia and suppress the ischemia-induced increase in c-Fos expression and apoptosis of the hippocampal CA1 region in gerbils [18, 19]. Xu et al. found that acupuncture and EA at the Baihui and Zusanli acupoints can not only reduce infarct size in the brain but also improve neurological function through neuronal protection [20]. Stem-cell-like cells have been found in adult brains, indicating that adult neuronal cells in brain tissue may regenerate [21]. Astrocytes, which are located at the center of the neurovascular unit and are responsible for the transfer of information between the synapses of neural cells, play an important role in the recovery of neuronal function and regeneration after ischemic stroke by providing energy support and mediating the activation and maturation of neuronal stem cells and the formation of synapses [22–25].

Some studies have investigated the effects of EA intervention in the MCAo model. Notably, EA at the Baihui acupoint may facilitate the recovery of motor function and stimulate brain-derived neurotrophic factor (BDNF) and receptor tyrosine kinase B expression in rats with cerebral ischemia [26]. Kim et al. demonstrated that EA pretreatment at the Baihui and Dazhui (GV14) acupoints may increase the production of BDNF and stromal cell-derived factor-1*α*, eliciting protective effects against focal cerebral ischemia [27]. Moreover, Yang et al. showed that EA can improve not only neuronal regeneration and newborn neuron migration but also the maturation of newborn neurons in the striatum of adult rat brains after stroke [28]. Tao et al. proposed that the upregulatory effect of EA on the notch signaling pathway and neurotrophic factor secretion may lead to hippocampal neural stem cell proliferation and a therapeutic effect on cerebral ischemia [29]. Microglia is reported as the major cell in CNS repair and regeneration [30]. Neural regeneration underlying brain tissue repair is also suggested to be involved in stroke [31]. Current treatment has been focused on targeting neural regeneration [32]. Therefore, the present study investigated neuronal regeneration in EA-treated cerebral infarction rats.

## 2. Materials and Methods

### 2.1. Animals

Adult male Sprague–Dawley (SD) rats weighing between 250 and 350 g were purchased from BioLASCO Taiwan Co., Ltd., and raised in the Animal Center of China Medical University (CMU) in a 12-12-hour light-dark cycle environment. The room temperature was controlled at 25°C, and adequate food and water were provided. Animal use was approved by the Institutional Animal Care and Use Committee of CMU, and all animal protocols were performed in accordance with the* Guide for the Use of Laboratory Animals* (National Academy Press).

### 2.2. Establishment of the Transient MCAo Model

Before the establishment of the MCAo model, the right MCA was exposed through a cranial burr hole that was 2.5 mm lateral and 2.0 mm posterior to the bregma. The MCA blood flow was monitored using Laser Doppler flowmetry (DRT4, Moor Instruments Inc., Wilmington, USA), and was found to be more than 500 min/div before MCAo. Thereafter, the right common carotid artery (CCA) and internal carotid artery (ICA) were exposed through a neck midline incision, followed by the ligation of the pterygopalatine artery proximal to its branch under isoflurane anesthesia. A 3-0 nylon filament suture blunted at the tip by a flame and coated with poly-L-lysine (Sigma, USA) was inserted into the right external carotid artery through the CCA and advanced up to the ICA at a distance of 20–25 mm to block the origin of the right MCA; subsequently, the MCA blood flow was found to decrease to less than 100 min/div. After 15 minutes of blocked blood flow from the right MCA, the suture was removed slowly and reperfusion was allowed for 24 hours.

### 2.3. Rat Selection and Grouping

At 24 hours after reperfusion, the modified neurological severity score (mNSS) was assessed, and the rotarod test (RRT) was performed. The present study only included rats with mNSS ≥ 7, except for the normal group. A total of 60 male SD rats were randomly divided into the following five groups (12 rats each): (1) normal group, (2) control group, (3) sham group, (4) EA1 group, and (5) EA2 group.

In the normal group, the trachea and right carotid artery of the rats were exposed for 15 minutes after a neck midline incision. The incision was then sutured under isoflurane anesthesia, which was subsequently reinduced in the rats for 15 minutes on the first (24 hours), third, fifth, and seventh days postoperation. Prior to this second anesthesia induction, mNSSs were assessed and RRTs were performed on the first day (24 hours after operation) as well as 4th and 8th days postoperation.

The methods applied to the control group were identical to those applied to the normal group; however, transient MCAo was performed. The methods applied to the sham group were identical to those applied to the control group; however, the acupuncture needles were inserted into the subcutaneous layer at both the Zusanli (ST36) and Shangjuxu (ST37) acupoints. Furthermore, the needles were connected to a stimulator (Trio 300, ITO Co., Ltd., Tokyo, Japan) without electrical discharge. The methods applied to the EA1 group were identical to those applied to the sham group, but the needles were inserted into the muscle layer at both the ST36 and ST37 acupoints. For this group, the needles were connected to the stimulator and received electrical stimulation at a frequency of 2 Hz; this produced muscle contractions that were visible slightly to the naked eye. Finally, the methods applied to the EA2 group were identical to those applied to the EA1 group; however, the electrical stimulation was applied at a frequency of 15 Hz.

All of the rats were sacrificed after completing mNSS and RRT on the 8th day after operation or reperfusion. Six rats from each group were used for the cerebral infarction size study, and the remaining six rats from each group were used for the immunohistochemistry (IHC) study.

### 2.4. mNSS Assessment and RRT

mNSS was assessed and RRT was performed on the first, fourth, and eighth day postoperation or reperfusion. mNSS was assessed by a well-trained investigator who was blinded to the groups. The scale provides a behavior deficit score, after reviewing motor, sensory, balance, and reflex functions; the total neurological deficit score is 18 [10, 33]. In the motor tests conducted in the present study, the rats were raised by their tails and received a score that ranged from 0 to 3; the rats were then placed on the floor and received a score that ranged from 0 to 3. For the sensory, beam balance, and reflex tests, the scores ranged from 0 to 2, 0 to 6, and 0 to 4, respectively.

The RRT apparatus (Rotamex, Columbus Instrument, Ohio, USA; picture 3.4) was composed of a striated rod (diameter: 8 cm) divided in five lanes (width: 5 cm) and located 8 cm above the ground. Each rat was trained for 3 days on a rotarod cylinder that accelerated from 4 to 40 rpm in 5 minutes, three times per day, and for an additional 5 minutes once before grouping. If a rat fell before 160 seconds, it was returned to the rotating rod for another training session until it reached the criterion [34]. The time taken for the RRTs performed three times per day was averaged and used as the RRT time.

The mNSS from the first day was used as the baseline. The rats were usually weak for the first 24 hours after reperfusion, which created a potential bias in the RRT time on the first day. Therefore, only the RRT times from the fourth and eighth days were used in the study.

### 2.5. Assessment of Cerebral Infarction Size

The rat brains were placed in a plastic rat brain model and were sectioned into six slices with 2 mm thickness from the frontal pole. The slices were then stained with 2% 2,3,5-triphenyl tetrazolium chloride (Merk, Germany) for 15 minutes. The area stained white was the ischemic infarction area, and the area stained purple–red was the nonischemic infarction area. After images of these areas were obtained, the third slice from the frontal pole was used to calculate the ratio of the ischemic area to the ipsilateral hemisphere area (I/H ratio) using Image J (USA).

### 2.6. IHC of Ki67, Glial Fibrillary Acidic Protein, and Nestin

Under deep isoflurane anesthesia, the rats were sacrificed after completing mNSS assessment and RRT on the eighth day after reperfusion. Subsequently, the rats were perfused transcardially with 0.9% sodium chloride and 4% paraformaldehyde for fixation. Their brains were removed and further fixed for 3 days in 4% paraformaldehyde at 4°C, followed by cryoprotection in 30% sucrose for 4 days at 4°C. Next, the brains were sliced into 300 *μ*m thick tissue sections in an optimum cutting temperature compound, and the frozen brain sections were cut into 20 *μ*m thick slices by using a Leica CM 3050 cryostat (Leica Microsystes, Wetzlar, Germany).

For the IHC staining, the brain slices were stained with antibodies against Ki67 (1 : 300; Millipore, USA), glial fibrillary acidic protein (GFAP; 1 : 200; Calbiochem, USA), and nestin (1 : 200; Millipore). The tissue was washed with phosphate buffered saline (PBS) and was then cocultured with 3% H_2_O_2_/methanol for 15 minutes. Next, the tissue was rewashed with PBS and was then cocultured with 10% normal blood serum for 20 minutes (LsAB kit, Zymed, San Francisco, CA, USA). Subsequently, blood serum was wiped away, and the tissue was cocultured with the primary antibody overnight, before being rewashed again with PBS three times and cocultured with the secondary antibody for 10 minutes. Thereafter, the tissue was washed with PBS three times. Finally, the tissue was cocultured with the Ls-AB-peroxidase complex for 10 minutes and then cocultured with DAB for 2 minutes (Liquid DAB substrate kit, Zymed). After hematoxylin staining, the glass slide was mounted for further observation. The stained slices were sealed under the coverslips and examined for the presence of immunoreactive cells using a microscope (Olympus, BX-51, Japan). The number of immunoreactive cells was quantified using National Institutes of Health ImageJ software (Bethesda, MD, USA).

### 2.7. Statistical Analyses

The data are presented as mean ± standard deviation. Between-group comparisons were performed using one-way analysis of variance, followed by Tukey's test. A *p* value of <0.05 was considered statistically significant.

## 3. Results

### 3.1. Effect of EA on Cerebral Infarct in Ischemia–Reperfusion-Injured Rats

**Figure 1:**
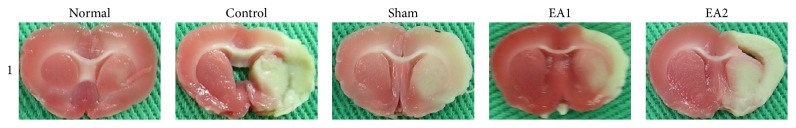
*Effect of electroacupuncture (EA) on cerebral infarction in ischemia–reperfusion-injured rats*. The ratios of ischemia/hemisphere in the control, sham, and EA2 groups were higher than that in the EA1 group. Normal: normal group; Control: control group; Sham: sham group; EA1: 2 Hz EA treatment group; EA2: 15 Hz EA treatment group; white color: ischemic infarction area; purple–red color: nonischemic infarction area.

The I/H ratios were higher in the control, sham, EA1, and EA2 groups than in the normal group on the eighth day after reperfusion (all *p* < 0.001; Table 1, Figure 1, *n* = 6). The I/H ratios were similar between the two groups among the control, sham, and EA2 groups on the eighth day after reperfusion (*p* > 0.05; Table 1, Figure 1, *n* = 6). Additionally, the I/H ratios were higher in the control, sham, and EA2 groups than in the EA1 group (all *p* < 0.05; Table 1, Figure 1, *n* = 6).

### 3.2. Effect of EA on mNSS in Ischemia–Reperfusion-Injured Rats

The mNSSs were higher in the control, sham, EA1, and EA2 groups than in the normal group on the first day after reperfusion (all *p* < 0.001; Table 1, *n* = 12). However, the mNSSs were similar between the two groups among the control, sham, EA1, and EA2 groups on the first day after reperfusion (all *p* > 0.05; Table 1, *n* = 12). This result indicated that the baseline was similar among the control, sham, EA1, and EA2 groups.

The mNSSs were also higher in the control, sham, EA1, and EA2 groups than in the normal group on the fourth day after reperfusion (all *p* < 0.001; Table 1, *n* = 12). Moreover, the mNSSs were higher in the control and sham groups than in the EA1 and EA2 groups on the fourth day after reperfusion (all *p* < 0.05; Table 1, *n* = 12). By contrast, the mNSSs were similar between the control and sham groups on the fourth day after reperfusion (*p* > 0.05; Table 1). The mNSS also was similar in the EA1 group and in the EA2 group on the fourth day after reperfusion (*p* > 0.05; Table 1, *n* = 12).

On the eighth day after reperfusion, the mNSSs were still higher in the control, sham, EA1, and EA2 groups than in the normal group (all *p* < 0.001; Table 1, *n* = 12). Moreover, the mNSSs were higher in the control and sham groups than in the EA1 and EA2 groups on the eighth day after reperfusion (all *p* < 0.001; Table 1, *n* = 12), but were similar between the control and sham groups on the same day (*p* > 0.05; Table 1, *n* = 12). Finally, the mNSS was higher in the EA2 group than in the EA1 group (*p* < 0.05; Table 1, *n* = 12).

### 3.3. Effect of EA on RRT Time in Ischemia–Reperfusion-Injured Rats

The RRT times were higher in the normal group than in the control, sham, EA1, and EA2 groups on the fourth day after reperfusion (all *p* < 0.001; Table 1, *n* = 12). Moreover, the RRT times were higher in the EA1 and EA2 groups than in the control and sham groups on the fourth day after reperfusion (all *p* < 0.05; Table 1, *n* = 12) and were similar between the control and sham groups on the same day (*p* > 0.05). Additionally, the RRT times were higher in the EA1 group than in the EA2 group on the fourth day after reperfusion (*p* < 0.05; Table 1, *n* = 12).

The RRT times were higher in the normal group than in the control, sham, EA1, and EA2 groups on the eighth day after reperfusion (all *p* < 0.001; Table 1, *n* = 12). The RRT times were also higher in the EA1 and EA2 groups than in the control and sham groups on the eighth day after reperfusion (all *p* < 0.05; Table 1, *n* = 12) and were similar between the control and sham groups on the same day (*p* > 0.05, *n* = 12). Finally, the RRT times were higher in the EA1 group than in the EA2 group on the eighth day after reperfusion (*p* < 0.05; Table 1, *n* = 12).

### 3.4. Effect of EA on Ki67, GFAP, and Nestin Immunoreactive Cells in Ischemia–Reperfusion-Injured Rats

**Figure 2:**
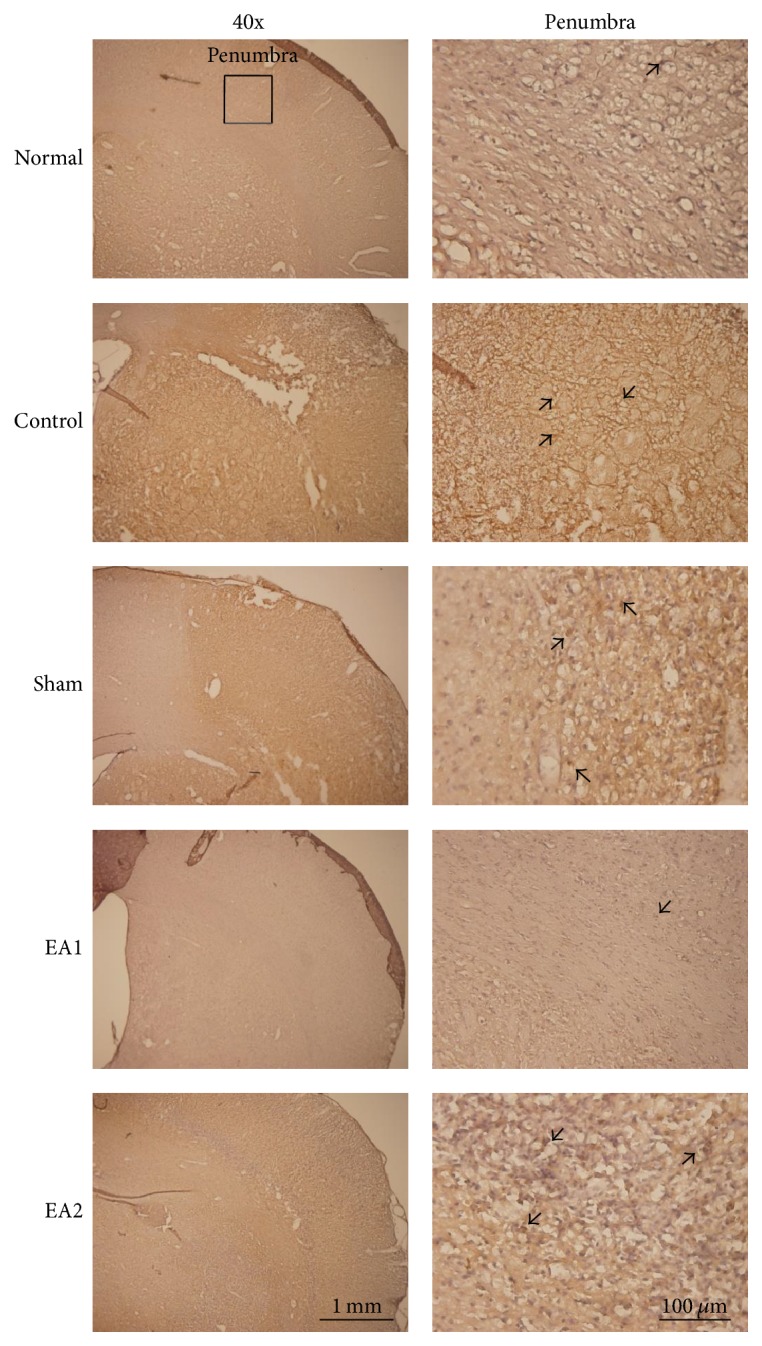
*Effect of EA on Ki67 immunoreactive cells in ischemia–reperfusion-injured rats*. The number of Ki67 immunoreactive cells increased in the control, sham, and EA2 groups in the penumbra area, but decreased in the EA1 group. Normal: normal group; Control: control group; Sham: sham group; EA1: 2 Hz EA treatment group; EA2: 15 Hz EA treatment group. Arrow means immunopositive cells.

In the penumbra area, the number of Ki67 immunoreactive cells in the control, sham, EA1, and EA2 groups was higher than that in the normal group (all *p* < 0.001; Table 2, Figure 2). The number of Ki67 immunoreactive cells in the control, sham, and EA2 groups was also higher than that in the EA1 group (all *p* < 0.05; Table 2, Figure 2), and the number of Ki67 immunoreactive cells in the control group was higher than that in the sham group (*p* < 0.05; Table 2, Figure 2). Furthermore, the number of Ki67 immunoreactive cells was similar between the control and EA2 groups (*p* > 0.05; Table 2, Figure 2).

**Figure 3:**
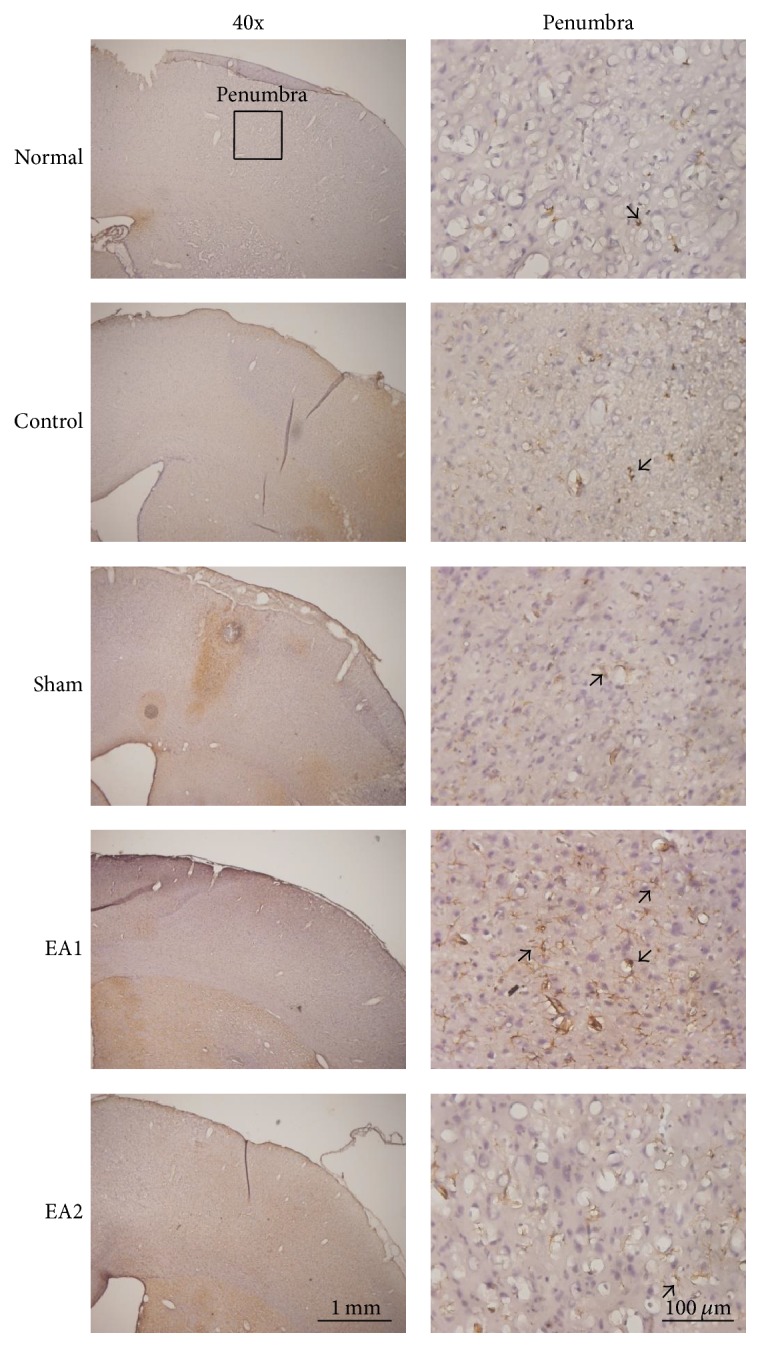
*Effect of EA on glial fibrillary acidic protein (GFAP) immunoreactive cells in ischemia–reperfusion-injured rats.* The number of GFAP immunoreactive cells was higher in the EA1 group that in the control, sham, and EA2 groups in the penumbra area. Normal: normal group; Control: control group; Sham: sham group; EA1: 2 Hz EA treatment group; EA2: 15 Hz EA treatment group. Arrow means immunopositive cells.

In the penumbra area, the number of GFAP immunoreactive cells in the control, sham, EA1, and EA2 groups was higher than that in the normal group (all *p* < 0.001; Table 2, Figure 3). The number of GFAP immunoreactive cells in the EA1 group was higher than that in the control, sham, and EA2 groups (all *p* < 0.05; Table 2, Figure 3); moreover, the number of GFAP immunoreactive cells was similar between the two groups among the control, sham, and EA2 groups (all *p* > 0.05; Table 2, Figure 3).

**Figure 4:**
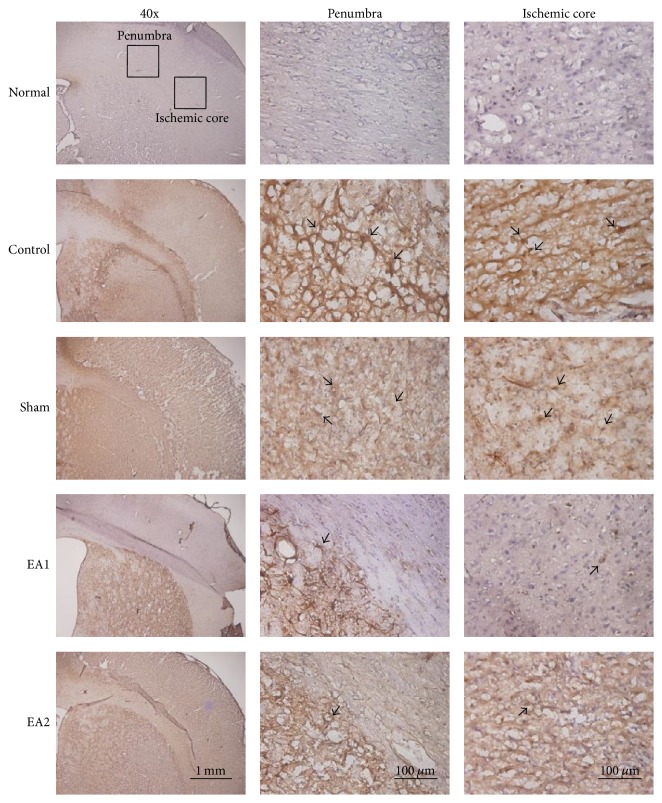
*Effect of EA on nestin immunoreactive cells in ischemia–reperfusion-injured rats*. The number of nestin immunoreactive cells increased in the control and sham groups in the penumbra and ischemic core areas, but decreased in those same areas in the EA1 and EA2 groups. Normal: normal group; Control: control group; Sham: sham group; EA1: 2 Hz EA treatment group; EA2: 15 Hz EA treatment group. Arrow means immunopositive cells.

In the penumbra area, no prominent nestin immunoreactive cells were noted in the normal group (Figure 4). Additionally, the number of nestin immunoreactive cells in the control and sham groups was higher than that in the EA1 and EA2 groups (all *p* < 0.05; Table 2, Figure 4). The number of nestin immunoreactive cells was also higher in the EA2 group than that in the EA1 group (*p* < 0.05; Table 2, Figure 4). Finally, the number of nestin immunoreactive cells was similar between the control and sham groups (*p* > 0.05; Table 2, Figure 4).

In the ischemic core area, no prominent nestin immunoreactive cells were noted in the normal group (Figure 4). The number of nestin immunoreactive cells in the control and sham groups was also higher than that in the EA1 and EA2 groups (all *p* < 0.05; Table 2, Figure 4). Furthermore, the number of nestin immunoreactive cells was higher in the EA2 group than in the EA1 group (*p* < 0.05; Table 2, Figure 4), and the number of such cells was similar between the control and sham groups (*p* > 0.05; Table 2, Figure 4).

## 4. Discussion

In the present study, the I/H ratios were higher in the control, sham, and EA2 groups than in the EA1 group on the eighth day after reperfusion. The mNSSs were higher in the control and sham groups than in the EA1 and EA2 groups on the fourth and eighth days after reperfusion. The RRT times were higher in the EA1 and EA2 groups than in the control and sham groups on the fourth and eighth day after reperfusion. Based on the aforementioned results, it was determined that 2 Hz EA could reduce cerebral infarction size and neurological deficit, whereas 15 Hz EA only improved neurological deficit in ischemia–reperfusion-injured rats. This suggests that 2 and 15 Hz EA have differential effects on cerebral infarct.

One previous study found that EA at the GV26 and PC6 acupoints could reduce cerebral infarction size in ischemia–reperfusion-injured rats [35]. EA at different acupoints produces various results regarding the reduction of cerebral infarction size; in other words, the protective effects of EA against cerebral ischemia are acupoint specific [16]. Wu et al. reported that acupuncture at the DU20, DU14, LI10, and ST36 acupoints effectively facilitated functional recovery and changed diffusion tensor imaging [36]. In our previous study, EA at both ST36 and ST37 acupoints increased the cerebral blood flow in rats with or without cerebral ischemia [37]. Additionally, EA at different frequencies triggers the release of distinct neuropeptides in the central nervous system (CNS) [38]. Wang et al. reported that EA at different frequencies might affect diverse pathways, resulting in various gene expression; notably, more genes were found to be differentially regulated by 2 Hz EA than by 100 Hz EA [39]. Taken together, 2 and 15 Hz EA exert partially different effects on cerebral infarction, possibly because of their different mechanisms. Additional studies should investigate the mechanisms underlying 2 and 15 Hz EA.

In the present study, 2 Hz EA also reduced the number of Ki67 immunoreactive cells and increased the number of GFAP immunoreactive cells in the penumbra region. However, 15 Hz EA could not alter immunoreactivity in the ischemia–reperfusion-injured rats. Ki67 is a nuclear protein that is used as a mitotic marker. Ki67 is also used as a proliferation marker and is expressed in the initial stage of mitosis during adult neurogenesis [40]. GFAP is an astrocyte-specific intermediate filament (IF) protein [41], and it is used as a biomarker to identify astrocytes in the CNS [42]. Nawashiro et al. established a permanent MCAo model and found that GFAP null mice had a larger cortical infarct volume, suggesting that astrocytes play a critical role in regulating the local cerebral blood flow in the penumbra region after ischemia [41]. Reactive astrocytes play a neuroprotective role after cerebral ischemia, given that astrocytes enhance glutamate transport after ischemia [43]. Moreover, reactive gliosis and glial scars are generated after an ischemic stroke in humans, and ablation of the reactive astrocytes increases the lesion size and leads to tissue damage in mice models, suggesting the beneficial role of gliosis. In addition, glial scars may function as a protective barrier against infectious agents and inflammatory cell invasion [44].

Nestin is not only a cytoplasmic intermediate filament (IF) protein associated with IF polymerization and macromolecule stability, but also identified in multipotent stem/progenitor cells in the CNS. In addition, nestin is normally expressed during CNS development and is reactivated after minor stresses to the nervous system [45]. During severe stresses, such as lesions involving glial scarring, nestin is upregulated. Because this upregulation lasts for up to 13 months postinjury, nestin upregulation may be associated with CNS glial scarring [45]. Moreover, nestin is found around neural stem cells and immature astrocyte glial cells during the embryonic period; after birth, however, no nestin is found around mature astrocyte glial cells, although it reappears around reactive astrocyte glial cells.

CNS injuries first induce nestin expression in reactive astrocyte glial cells near the wound and then lead to hyperplasia, deformation, transition, and scarring of reactive astrocyte glial cells, sequentially [46]. Shin et al. found that astrocytes disappear in the ischemic core area and that the induction of nestin expression is only associated with vasculature including microvasculature and larger caliber vessels. In the ischemic core area, the induction of nestin expression occurs 3 days after ischemia and continues for at least 28 days after ischemia; however, the cellular construction of nestin positive cells changes during this period. Therefore, nestin expression is not only limited to the progenitor/stem cell population, but also associated with vasculature-associated cells in the ischemic core area [47]. Taken together, EA at ST36 and ST37 plays a neuroprotective role in ischemia–reperfusion-injured rats.

The present study has some limitations to acknowledge. The baseline was set only according to the mNSSs at 24 hours after reperfusion, which could not explain whether the cerebral infarction size was similar among the control, sham, EA1, and EA2 groups. Instead, imaging studies involving computer tomography or magnetic resonance imaging should be used to detect the infarction size in live animals.

## 5. Conclusion

Treatment with 2 Hz EA at ST36 and ST37 acupoints was determined to reduce cerebral infarction size and neurological deficit scores, as well as increase RRT times. In addition, 2 Hz EA reduced Ki67 and nestin immunoreactive cells and increased GFAP immunoreactive cells in ischemia–reperfusion-injured cerebral infarction rats. These findings suggest that EA plays a neuroprotective role in ischemia–reperfusion-injured rats, although the underlying mechanisms require further study.

## Figures and Tables

**Table 1:** Effect of electroacupuncture (EA) on cerebral infarct and neurological deficits in ischemia–reperfusion-injured rats.

	Group				
	Normal	Control	Sham	EA1	EA2
I/H ratio	0.00 ± 0.00	0.73 ± 0.05^*∗*^	0.72 ± 0.04^*∗*^	0.23 ± 0.19^*∗*#¶†^	0.70 ± 0.06^*∗*^

mNSS					
Day 1	0.0 ± 0.0	7.4 ± 0.7^*∗*^	7.2 ± 0.4^*∗*^	7.1 ± 0.3^*∗*^	7.1 ± 0.3^*∗*^
Day 4	0.0 ± 0.0	6.7 ± 0.8^*∗*^	6.5 ± 0.5^*∗*^	4.3 ± 0.9^*∗*#¶^	4.8 ± 0.9^*∗*#¶^
Day 8	0.0 ± 0.0	6.4 ± 0.7^*∗*^	6.2 ± 0.8^*∗*^	3.2 ± 0.7^*∗*#¶†^	4.2 ± 0.8^*∗*#¶^
RRT					
Day 4	169.1 ± 20.9	41.8 ± 13.7^*∗*^	58.4 ± 12.8^*∗*^	126.7 ± 38.3^*∗*#¶†^	91.5 ± 22.1^*∗*#¶^
Day 8	183.3 ± 13.9	49.8 ± 15.9^*∗*^	64.3 ± 18.7^*∗*^	156.7 ± 46.0^*∗*#¶†^	117.0 ± 23.7^#¶^

Data represent mean ± standard deviation. Normal: normal group; Control: control group; Sham: sham group; EA1: 2 Hz electroacupuncture treatment group; EA2: 15 Hz electroacupuncture group; I/H: infarction/hemisphere ratio; mNSS: modified neurological severity score; RRT: rotarod test; ^*∗*^*p* < 0.001 compared with Normal; ^#^*p* < 0.05 compared with Control; ^¶^*p* < 0.05 compared with Sham; ^†^*p* < 0.05 compared with EA2; Day 1: 24 hours after reperfusion; Day 4: four days after reperfusion; Day 8: eight days after reperfusion; repeated measures analysis of variance, followed by Tukey's test.

**Table 2:** Effect of EA on Ki67, GFAP, and nestin immunoreactive cells in ischemia–reperfusion-injured rats.

	Group				
	Normal (*N* = 6)	Control (*N* = 6)	Sham (*N* = 6)	EA1 (*N* = 6)	EA2 (*N* = 6)
Ki67 (P)	10.2 ± 11.4^*∗*^	122.0 ± 14.8^*∗*^	96.5 ± 13.2^*∗*#^	47.0 ± 7.7^#*∗*¶†^	110.0 ± 17.5^*∗*^
GFAP (P)	16.5 ± 10.8	55.2 ± 16.4^*∗*^	49.8 ± 11.6^*∗*^	105.8 ± 13.8^*∗*#¶†^	58.3 ± 8.5^*∗*^
Nestin (P)	0.0 ± 0.0	290.7 ± 18.8^*∗*^	283.7 ± 23.2^*∗*^	92.3 ± 8.9^*∗*#¶†^	219.7 ± 23.7^*∗*#¶^
Nestin (IC)	0.00 ± 0.0	409.0 ± 37.5^*∗*^	395.2 ± 18.5^*∗*^	181.7 ± 21.9^*∗*#¶†^	281.3 ± 22.5^*∗*#¶^

Data represent mean ± standard deviation. Normal: normal group; Control: control group; Sham: sham group; EA1: 2-Hz electroacupuncture treatment group; EA2: 15-Hz electroacupuncture group; Ki67: Ki67 immunoreactive cells; GFAP: GFAP immunoreactive cells; Nestin: nestin immunoreactive cells; P: penumbra area; IC: ischemic core area; ^*∗*^*p* < 0.001 compared with Normal; ^#^*p* < 0.05 compared with Control; ^¶^*p* < 0.05 compared with Sham; ^†^*p* < 0.05 compared with EA2; repeated measures analysis of variance, followed by Tukey's test.
